# High co‐incidence of coeliac disease in paediatric type 1 diabetes: A call for systematic screening

**DOI:** 10.1111/dom.70395

**Published:** 2025-12-19

**Authors:** Giulio Maltoni, Luca Bernardini, Andrea Scozzarella, Egidio Candela, Giulia Montanari, Edoardo Petracci, Marcello Lanari

**Affiliations:** ^1^ Pediatric Unit IRCCS Azienda Ospedaliero‐Universitaria di Bologna Bologna Italy; ^2^ Department of Medical and Surgical Sciences, Alma Mater Studiorum University of Bologna Bologna Italy; ^3^ Specialty School of Pediatrics, Alma Mater Studiorum University of Bologna Bologna Italy

**Keywords:** coeliac disease, database research, observational study, real‐world evidence, screening, type 1 diabetes

## 1 BACKGROUND

Type 1 diabetes (T1D) and coeliac disease (CD) are closely associated autoimmune disorders that share common human leukocyte antigen‐related genetic and immunological pathways,^1^, ^2^ with additional environmental contributions.^3^, ^4^

Early and appropriate screening for CD is therefore crucial to ensure timely diagnosis. According to the latest guidelines of the International Society for Paediatric and Adolescent Diabetes (ISPAD), children with T1D should be screened for CD at diagnosis and, if negative, again at 2 and 5 years, because most CD diagnoses occur within 10 years of T1D onset, particularly during the first year.^5^

In the general population, the worldwide prevalence of CD is approximately 1.4%, although marked geographic variability exists.^6^ Incidence has increased steadily in most industrialised countries—especially Europe, North America and Oceania—while in other regions it has stabilised or declined. Italy is among the countries with the highest CD incidence in Europe.^7^ The prevalence of CD is substantially higher among youth with T1D. International registry data from North America, Europe and Oceania report an overall prevalence of 3.5%.^8^ In our region, previous studies showed an incidence of 3.3% among children with T1D diagnosed between 1987 and 1994, rising to 10.5% in the 1995–2004 cohort, indicating a progressive increase over time.^9^

The present study aimed to assess how this association has evolved in our paediatric cohort over the past 10 years through a retrospective analysis.

## 2 MATERIALS AND METHODS

We included 236 individuals (131 males and 105 females) under 18 years of age with new‐onset T1D between 1st June 2010 and 31st May 2020, with a minimum follow‐up of 5 years (until May 2025) at the Paediatric Diabetes Centre of Istituto di Ricovero e Cura a Carattere Scientifico Azienda Ospedaliero‐Universitaria di Bologna, a tertiary‐care referral hospital serving the Bologna metropolitan area and neighbouring provinces in the Emilia‐Romagna region (Northern Italy).

Data were retrospectively collected from our registry. To minimise referral bias (‘hotspot effect’), we included only children and adolescents whose diabetes onset occurred at our centre. All subjects underwent immunological evaluation at T1D onset and annually thereafter. Screening for CD consisted of measuring total Immunoglobulin A (IgA) and Anti–tissue transglutaminase immunoglobulin A (TGA‐IgA). When TGA‐IgA were ≥10 times the upper limit of normal, endomysial antibodies (EMA‐IgA) were subsequently assessed on a second blood sample to confirm the serology‐based diagnosis, according to the latest European Society for Paediatric Gastroenterology, Hepatology and Nutrition (ESPGHAN) recommendations. Most diagnoses were made according to the previous ESPGHAN criteria, which also required human leukocyte antigen typing and histological confirmation by gastroduodenoscopy.

Data on diabetic ketoacidosis (DKA) at T1D onset were available for all individuals. DKA was classified as mild/moderate or severe according to standard biochemical and clinical criteria.

Statistical analyses were performed using GraphPad Prism (Windows version). Results are expressed as mean ± standard deviation (SD) or as proportions (%). The *χ* ^2^ test was used to assess differences in proportions. Statistical significance was set at *p* < 0.05. Period‐prevalence was calculated as the number of new cases of CD within the study divided by the number of new cases of T1D diagnosed during the same period. The study was approved by the Ethics Committee of the Emilia Centro Area, Emilia‐Romagna Region (ESORD1T; 323/2020/OSS/AOUBo).

## 3 RESULTS

A total of 236 individuals with new‐onset T1D between 1st June 2010 and 31st May 2020 were included. The mean age at T1D onset was 7.44 ± 2.84 years. A minimum follow‐up of 5 years was required to evaluate the occurrence of CD. During this period, 46 subjects were diagnosed with both CD and T1D, corresponding to a 10‐year period‐prevalence of 19.5%. Of these, 25 were females (54.3%) and 21 males (45.6%). Most subjects (89.1%) were Caucasian.

**FIGURE 1:**
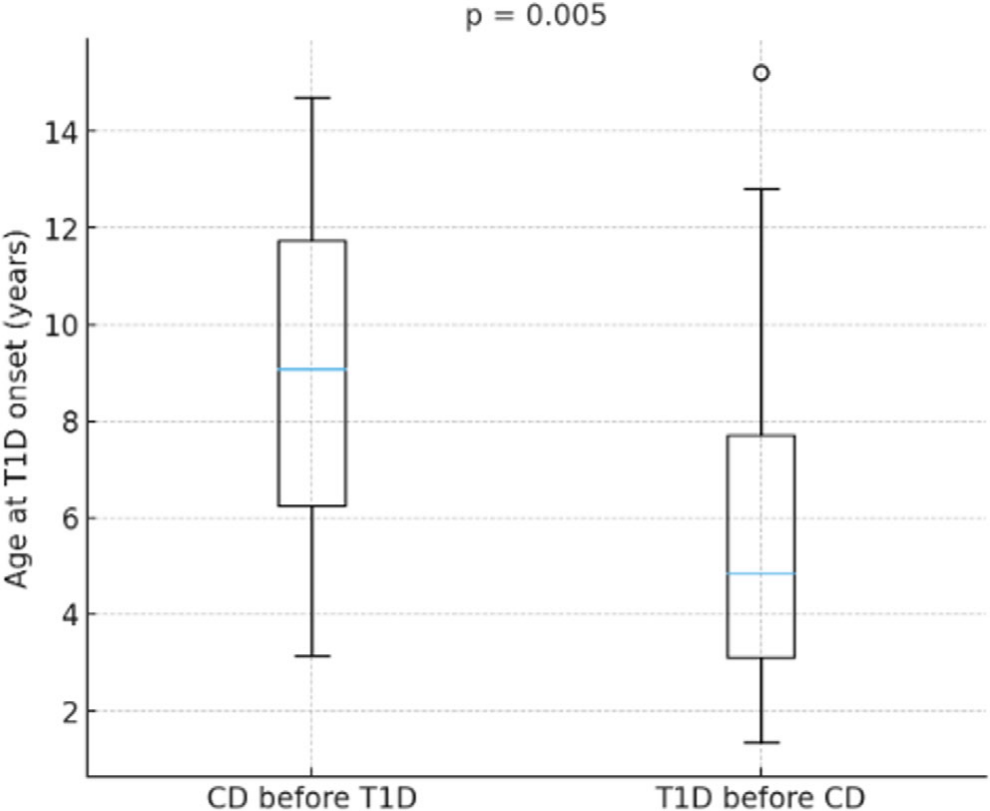
Distribution of age at type 1 diabetes (T1D) onset stratified by disease sequence. Boxes represent the median and interquartile range; whiskers extend to the most extreme values. The *p*‐value reflects the between‐group comparison (Mann–Whitney *U* test). CD, coeliac disease.

The mean age at T1D onset was 6.9 ± 3.8 years, while the mean age at CD onset was 5.9 ± 3.9 years (Figure 1).

Of the 46 subjects diagnosed with CD, 43.5% (*n* = 20/46) had anti‐tissue transglutaminase IgA values greater than 10 times the upper limit of normal, 56.5% (*n* = 26/46) underwent duodenal biopsy for diagnostic confirmation, while the remaining 20 patients (43.5%) received a serology‐based diagnosis without biopsy, consistent with ESPGHAN criteria.^10^ Finally, no cases of total IgA deficiency were identified among these 46 subjects.

Overall, 14 out of 46 individuals (30.4%) presented with DKA at T1D onset, including 5 severe cases (10.9%). When stratified according to the temporal relationship between CD and T1D onset, no statistically significant difference emerged across groups; however, a clear increasing trend in DKA frequency was observed: 3/20 individuals with a prior CD diagnosis (15%, one severe), 3/10 with simultaneous diagnoses (30%, one severe) and 8/16 who developed CD after T1D (50%, three severe).

No sex‐related differences were observed. Among the 46 youths with both diagnoses, CD was diagnosed after T1D in 16 cases (34.8%). In this subgroup, approximately 19.7 months passed between the mean age at onset of CD (6.84 ± 4.66 years) and the age at onset of T1D (5.20 ± 4.15 years). Describing the characteristics of the 20 individuals (43.5%) who make up the group in which CD preceded T1D, the mean age at onset of T1D (8.56 ± 3.40 years) was significantly higher than the age at onset of CD (4.91 ± 3.59 years). Finally, 10 children (21.7%) received both diagnoses simultaneously.

Overall, the age at onset of T1D was significantly lower in individuals who subsequently developed CD (5.20 ± 4.15 years) than in those in whom CD was diagnosed first (8.56 ± 3.40 years; *p* = 0.005). This difference between the groups is clearly illustrated in Figure 1, where the boxplot compares children who had a previous diagnosis of CD (‘CD before T1D’) with those who developed CD after T1D (‘T1D before CD’), showing a shift towards younger ages at diabetes onset in the ‘T1D before CD’ subgroup.

## 4 DISCUSSION

Our study demonstrates a notable increase in the cumulative incidence of CD among individuals with concomitant T1D in our region (19.5%), confirming a rising secular trend in the coexistence of these two autoimmune diseases. The simultaneous presence of CD should always be suspected in youths with T1D, especially given the significant impact that this second chronic condition can have both psychologically and in terms of greater difficulty in controlling euglycaemia.^11^, ^12^

According to the study by Pharm‐Short et al.,^13^ youths with T1D showed the highest risk of developing CD within the first 2 years after T1D onset, consistent with our data.

Differing from previous studies, we did not observe an earlier onset of T1D in children with CD. Conversely, the age at T1D onset was significantly lower in individuals who developed CD after T1D than in those in whom CD preceded diabetes. This finding supports the notion that T1D onset at preschool age itself represents a risk factor for the subsequent development of CD, particularly during the early years following T1D onset. Interestingly, a greater proportion of subjects in our cohort were diagnosed with CD before T1D, a finding not previously reported in similar studies. Notably, these children were already on a gluten‐free diet at the time of T1D diagnosis, which challenges the hypothesis that gluten restriction may be a protective factor against the onset of diabetes.

In our cohort, DKA at T1D onset was less common in children with a pre‐existing diagnosis of CD, and more frequent in those who were diagnosed with CD only after developing diabetes. Although this trend did not reach statistical significance, it is reasonable to assume that earlier referral and closer clinical surveillance may have facilitated a timelier recognition of diabetes symptoms in the former group. At present, the literature does not demonstrate a consistent influence of CD timing on the severity of diabetes onset,^14^, ^15^ and larger studies are needed to determine whether this observation represents a reproducible pattern rather than a centre‐specific finding.

Main limitations include its single‐centre retrospective design.

In conclusion, our findings suggest that individuals affected by either T1D or CD are more likely to develop the other condition than in previous decades. Preschool‐onset T1D remains a risk factor for subsequent CD development, particularly in the first years after T1D diagnosis. These findings highlight the need for systematic annual CD screening in paediatric T1D. In line with our findings, the implementation of the Type 1 Diabetes and Coeliac Disease (Italian National Screening Programme) in Italy (approved in 2023) underscores the importance of early and integrated screening for T1D and CD in paediatric populations.^16^

## FUNDING INFORMATION

This research received no specific grant from any funding agency in the public, commercial or not‐for‐profit sectors.

## CONFLICT OF INTEREST STATEMENT

The authors declare no conflicts of interest.

## ETHICS STATEMENT

The study was approved by the Ethics Committee of the Emilia Centro Area, Emilia‐Romagna Region (ESORD1T; 323/2020/OSS/AOUBo) and conducted in accordance with the Declaration of Helsinki. Written informed consent was obtained from the participants' parents or legal guardians.

## Data Availability

The data that support the findings of this study are available from the corresponding author upon reasonable request.
